
JOURNAL INFORMATION
==============================
NLM Title Abbreviation: ScientificWorldJournal
Journal ID: ScientificWorldJournal
NLM Journal ID: 101131163
Journal ID: TSWJ

The Scientific World Journal

ISSN: 2356-6140
EISSN: 1537-744X
Publisher: Wiley

ARTICLE INFORMATION
==============================
PMCID: PMC5956404
PMID: 15349561
DOI: 10.1100/tsw.2004.93
Article ID: 476428
Article version: 1
Subjects: Research Article

Radiation Therapy After Radical Prostatectomy

Ziada Ali M.
Crawford E. David
Division of Urology, University of Colorado, Denver, CO, USA
Academic Editor: Anthony Atala

Publication date: 2004
Electronic publication date: 2004 Jun 7
Volume: 4
First page: 377
Last page: 381
Copyright: Copyright © 2004 Ali M. Ziada and E. D. Crawford.
Copyright year: 2004
License: This is an open access article distributed under the Creative Commons Attribution License, which permits unrestricted use, distribution, and reproduction in any medium, provided the original work is properly cited.
License URL: https://creativecommons.org/licenses/by/3.0/

==============================
Articles
*TheScientificWorldJOURNAL* (2004) 4 (S1), 377–381
ISSN 1537-744X; DOI 10.1100/tsw.2004.93

### Radiation Therapy After Radical Prostatectomy

Ali M. Ziada, M.D. and E. David Crawford, M.D.
*Division of Urology, University of Colorado, Denver, Colorado*

**E-mails**: aziada@mednet3.camed.eun.eg

*Previously published in the Digital Urology Journal*

**DOMAIN**: urology

With the current advances in screening, earlier detection of prostate cancer, and improved techniques for performing radical prostatectomy, a larger number of patients are being surgically treated. However, on pathologic examination of their prostate, these patients might not have organ confined disease. The pathologic features which predict failure in those cases are unknown prior to surgery. This includes patients with seminal vesicle invasion, positive surgical margins, and high Gleason scores. Patients at high risk for local recurrence are candidates for further therapy to control the local disease. These patients represent a large proportion of surgically treated patients. In spite of 58% of patients having clinically localized disease at presentation, over one-half will likely be T3 disease.[1] This is despite all the advances in screening.

Adjuvant radiotherapy and/or various methods of androgen deprivation are available for this group of patients. A Mayo Clinic study found that 5-year local recurrence-free survival rates were >95% among prostatectomy patients who had either radiation or orchiectomy compared with 84% for patients who received no adjuvant therapy.[2] However, no advantage for adjuvant therapy was found in overall or cause-specific mortality. One recent study reported the efficacy of adjuvant radiation after radical prostatectomy, but only among patients found with pT3 who had an undetectable PSA after surgery.[3]

Postoperative radiation therapy can be given as adjuvant or salvage irradiation. Adjuvant radiation therapy can be used postoperatively for patients with high risk of recurrence. Radiation used as salvage therapy is usually associated with biochemical failure, whether it is associated with a palpable local or biopsy-detected lesion or not. The questions that we need to answer are:

- Does radiation therapy following surgery offer any advantage in local control?
- Does radiation therapy in this setting have any influence on survival and mortality?
- What predicts patients prognosis after radical prostatectomy?
- What should our indications be to use radiation therapy in the postoperative setting?
- How can radiation therapy be used most effectively -- salvage or adjuvant?

### EFFICACY OF RADIATION IN THE POSTOPERATIVE SETTING

Men with pathological stage T3 can be further subdivided into prognostically distinct subgroups based on multiple histologic and biochemical factors. Historically, a number of studies with long term follow up showed that extracapsular disease predisposes local failure. Gibbons et al[4] had 45 patients with microscopic extension beyond the prostatic capsule. Twenty-two of those patients received external beam irradiation therapy whereas 23 did not. Local recurrence rates (4%) were reduced in the group that received the radiation compared to 30% in the other group that did not. Survival, however, was not affected in either group. Radiation therapy was associated with a higher incidence of pelvic complications. The significance of this finding is of minor importance because of the more advanced techniques of radiation therapy used in recent years. Another study by Shevlin et al[5] showed that the local recurrence rate can be reduced from 28% to less than 5% with the use of adjuvant radiation.

A more recent study in 1993 by Cheng et al[2] found that following prostatectomy, the 5 year survival rate was more than 95% in patients receiving radiation or orchiectomy as adjuvant treatment, compared with 84% for other patients not receiving any form of adjuvant therapy. Again, development of distant metastasis and survival were not affected by the adjuvant radiation therapy.

In a recent series,[6] it was shown that a similar proportion of patients (77%) who received radiation therapy were free of metastatic disease, in comparison with patients (66%) who did not receive radiation. Multiple studies have shown that salvage radiation therapy has variable short term effects on the biochemical status of prostate cancer patients. The probability of developing rising PSA levels after prostatectomy ranges from 18% to 51%, and the duration of undetectable serum PSA is generally short averaging 16 to 24 months in different studies.[7]

Since rising PSA in modern reports is considered the primary indication of failure (local failure or distant metastasis or even both), only those with local failure will benefit from adjuvant radiation therapy. In a recent study by Forman et al,[8] patients with rising PSA received definitive radiotherapy. Sixty-four percent of patients exhibited no evidence of recurrence up to 48 months after therapy. Treatment failure was 3.7 times greater for patients who did not reach undetectable PSA levels, and 5.6 times greater for patients with either lymph node or seminal vesicle involvement. Patients who had a PSA >1.2 - 2.0 ng/ml postoperatively had a 4 to 5 times greater risk of failing salvage radiation than patients who had PSA values below this mark.

### PATTERNS OF FAILURE FOR PT3 PATIENTS

Several studies pointed out that patients with extracapsular disease are predisposed to failure following radical prostatectomy. The local failure rates of Gibbons et al,[4] Shevlin et al,[5] and Schellhammer[9] were 25%, 28% and 31% respectively. Myers and Fleming11 reported a 46% incidence of local recurrence after 10 years. Gibbons et al and Schellhammer suggested that local failure can be predicted by seminal vesicle involvement.

In a multivariate analysis study by Anscher and Prosnitz,[10] the most important predictors of local recurrence were positive surgical margins and high tumor grade. Capsular penetration alone was not a highly suggestive factor. In this same multivariate analysis, seminal vesicle involvement was found to be the strongest predictor of distant metastasis. However, seminal vesicle involvement was not an independent predictor of local failure.[10] The incidence of seminal vesicle involvement with local failure may be higher than reported, but is masked by the fact that patients do not survive due to distant metastasis or the institution of androgen suppression.

### Positive Surgical Margins

By strict definition, "positive margins" means neoplastic cells in contact with the inked margin used to mark the specimen. To clarify the positive margin phenomenon, 3 different categories were recognized by Sakr and Grignon:[12]

- Tumor exceeding the inked margin of resection of a seemingly intact anatomical boundary of the gland. In this incidence, local recurrence rates will be similar to organ confined disease.
- Tumor that extends to an irregular, shattered margin indicating surgical induced positive margin.This is commonly a finding at the prostatic apex.
- Tumor extending to definitive extraprostatic fat and/or soft tissue. This is clearly stage T3 cancer.

According to this refined classification by Sakr and Grignon,[12] patients with tumor involving surgical margins but without extraprostatic extension experience biochemical failure rates, in the form of rising PSA, between those of patients with confined tumors and those with extraprostatic disease. Also according to this classification, PSA failure is related to the degree of involvement of the surgical margin.

Patients with positive margins at radical prostatectomy have a 42-64% probability of remaining progression free at 5. This is in contrast to the 80% probability of patients with negative margins remaining progression free at 5 years after radical prostatectomy.[13,14]

Two studies relating progression to positive margins have followed patients for 10 years. In one of those studies, patients with negative margins had a progression free probability of 74.8%, while those with positive margins had a progression free probability of only 41.9%.[13] In the other study, progression free probability for patients with positive margins was 38%. Negative margins were not evaluated because these patients were in only 2 separate groups: those with organ confined disease and those with capsular penetration.[15]

However, studies found that positive surgical margin was predictive of recurrence only when Gleason sum equal to or greater than 6 with negative seminal vesicle and nodal involvement.[14] In another report, margin status is most relevant in patients with Gleason scores of 5 to 7. In men with Gleason scores 2 to 4, negative seminal vesicles, and negative lymph nodes, prognosis is known to be extremely favorable, while in patients with Gleason scores of 8 or 9, tumors cannot be stratified by capsular penetration or margin status.[16]

Huland et al[17] found that in nearly 50% of patients with a Gleason sum of 5 or 6, tumors were organ confined with negative margins. These patients had a 98.7% chance of being disease free at five years, and a 92.4% chance at 10 years, respectively. Since 88% of prostate cancer patients with negative seminal vesicles and nodal involvement fall into this category of a Gleason sum between 5 and 7, the surgical margin status would be of great value in determining the patients' chance of remaining disease free.[13,16]

Seminal vesicle invasion or lymph node involvement carries a 5 year progression rate of 31% and 23%, respectively. At 10 years, this rises to 58% and 55% respectively. Hence, seminal vesicle invasion or lymph node metastasis patients should be candidates for adjuvant therapy, whether radiation or hormonal. If these findings are detected preoperatively, they should not be candidates for surgery as a means of local control.

### IMMEDIATE ADJUVANT OR SALVAGE RADIATION

Historical studies suggest that waiting until gross local recurrence occurs is too late a stage for any local therapy, suggesting it would be more difficult to control gross recurrence versus microscopic residual disease. The Massachusetts General Hospital (MGH) data[18] published in 1993 showed that fewer than 40% of patients irradiated for palpable local recurrences were biochemically disease free at 2 years. The clinical free survival rates were less than 20% at 10 years following radiation therapy. Total "cure" chances are highly unlikely.

However, it is the policy in many institutions to not give radiation to those at risk of local recurrence but rather to wait for signs of biochemical failure. The rationale is that these patients may have no disease or, on the other hand, may have occult metastatic disease. In both cases, they will not benefit from local radiation therapy and will be exposed to the risk of side effects, even though it was reported that patients with occult distant metastasis may briefly benefit from radiation.[19] The results of delayed radiation were also inaccurate due to the fact that some studies included patients with distant metastasis. When lymph node involvement was excluded, the results were up to 48% freedom from biochemical recurrence at 3 years[20] and as high as 68% 3 year freedom from biochemical failure if seminal vesicle invasion was also excluded.[21] Salvage irradiation also showed some positive results. In contrast to the previous results elsewhere, data from Johns Hopkins Hospital show that 20% of patients were successfully salvaged.[22] Nearly half of these patients, however, had seminal vesicle invasion, nodal metastasis, or were at greater risk for distant rather than local recurrence.

The evidence is conflicting regarding the timing of radiation therapy. Long term studies suggest an advantage when adjuvant radiation is given compared to salvage radiation later on. However, this data was obtained prior to the PSA era. Using PSA in follow up means earlier detection of failure. According to Schild et al,[20] PSA suppression is inversely related to the PSA level at the time of radiation therapy. This also correlates with the Forman et al data previously discussed in this article. PSA follow up allows us to detect early failure, and hence radiation therapy can be given when tumor volume is small. The critical PSA level was 1.1 ng/ml in the Schild et al study. It is also important that the chance of benefiting from radiation therapy is much higher in delayed rise of PSA beyond the first year of follow up. This means that follow up should be strict and close. If this is not possible or available, early adjuvant radiation may be a reasonable option. Patients who had a PSA >1.2 or 2.0 ng/ml postoperatively had a 4 to 5 times greater risk of failing salvage radiation than patients who had PSA values below this mark.

Radiation's optimal dose varies according to the estimate of tumor burden. The USC group[23] used radiation doses less than those employed by MGH.[18] USC dosage was 45 to 50 Gy versus MGH's 60 to 64 Gy. In the Schild et al study, a dose of 64 Gy was found to be more effective than lower doses, with little morbidity.

### CONCLUSION

The main criticism of adjuvant radiation has been its failure to offer survival advantages. This may be due to other competing morbidities masking the survival advantage. Another hypothesis is that the poor biological response of the tumor tends to lead to local recurrence.

The issue of effectiveness of adjuvant radiation still awaits the results of a large Southwest Oncology Group (SWOG) trial. The current SWOG trial examines the benefits of adjuvant XRT with positive margins and capsular penetration after radical prostatectomy. The study randomizes men to adjuvant radiation therapy or to observation. Other data shows the potential benefit with little risk which should not be denied to patients with considerable risk for local recurrence. The patients least likely to benefit from adjuvant or salvage radiation are those with persistently elevated PSA, seminal vesicle invasion, or poorly differentiated tumors. Ultrasound biopsies of the anastomotic site may be helpful to document local recurrence, but negative results should not exclude local disease.

ProstaScint, which is a radiolabeled monoclonal antibody directed towards the prostate specific membrane antigen (PSMA), can be very promising in evaluating patients with rising PSA post radical prostatectomy as well as detection of pelvic lymph node metastasis. PSMA is found in greater amounts in malignant cells and in metastatic deposits, and is also expressed in 95% of all prostate cancer tissues studied. It can very useful in further determining which patients will benefit from post-operative radiation if there is local residual disease.[24]

### REFERENCES

1. Wingo PA, Tong T, Bolden S. Cancer statistics. CA Cancer J Clin. 1995;45:8-30.
2. Cheng WS, Frydenberg M, Bergstralh EJ, Larson-Keller JJ, Zincke H. Radical prostatectomy for pathological stage C prostate cancer: influence of pathologic variables and adjuvant treatment on disease outcome. Urology 1993; 42: 283-291.

3. Pilepich MJ, Krall JM, Al-Sarraf M, et al. Androgen deprivation with radiation therapy compared with radiation therapy alone for locally advanced prostatic carcinoma: a randomized comparative trial of the radiation therapy oncology group. Urology. 1995;45:616-623.
4. Gibbons RP, Cole BS, Richardson RG, et al. Adjuvant radiotherapy following radical prostatectomy: results and complications. J Urol 1986; 135: 65-68.
5. Shevlin BE, Mittal BB, Brand WN, Shetty RM. The role of adjuvant radiation following primary prostatectomy, based on histopathologic extent of tumor. Int J Radiat Oncol Biol Phys 1989; 16: 1425-1450.
6. Syndikus I, Pickles T, Kostashok E, Sullivan LD. Postoperative radiotherapy for stage pT3 carcinoma of the prostate; improved local control. J Urol 1996; 155, 1983-1986.
7. Andriole GL. Adjuvant therapy for prostate cancer patients at high risk of recurrence following radical prostatectomy. Eur Urol 1997; 32(suppl 3): 65-69.
8. Forman JD, Pontes D, Wood D Jr, et al. Therapeutic irradiation for patients with an elevated post-prostatectomy PSA level. Int J Radiat Oncol Biol Phys. In Press.
9. Schellhammer PF. Radical prostatectomy: patterns of local failure and survival in 67 patients. Urology 1988; 31: 191-197.
10. Anscher MS, Prosnitz LP. Multivariate analysis of factors predicting local relapse after radical prostatectomy-possible indications for postoperative radiotherapy. Int J Radiat Oncol Biol Phys 1991; 21: 941-947.
11. Myers RP, Fleming TR. Course of localized adenocarcinoma of the prostate treated by radical prostatectomy. Prostate 1983; 4: 461-472.
12. Sakr WA, Grignon DJ. Prostate cancer: indicators of aggressiveness. Eur Urol 1997; 32 (suppl 3): 14-23.
13. Epstein JI, Partin AW, Sauvageot J, et al. Prediction of progression following radical prostatectomy: a multivariate analysis of 721 men with long term follow up. Am J Surg Pahtol 1996; 20: 286-292.
14. Ohori M, Wheeler Tm, Kattan MW, et al. Prognostic significance of positive surgical margin in radical prostatectomy specimens. J Urol 1995; 154: 1818-1824.
15. Paulson DF. Impact of radical prostatectomy in the management of clinically localized disease. J Urol 1994; 152: 1826-1830.
16. Epstein JI. Incidence and significance of positive margins in radical prostatectomy specimens. Urol Clin North Am 1996; 23: 651-663.
17. Huland H, Hammerer P, Henke RP, Huland E. Preoperative prediction of tumor heterogeneity and recurrence after radical prostatectomy for localized prostatic carcinoma with digital rectal examination, PSA, and the results of six systemic biopsies. J Urol 1996; 155: 1344-1347.
18. Zietman AL, Coen JJ, Shipley WU, Althausen AF. Adjuvant irradiation following radical prostatectomy: analysis of freedom from PSA failure. Urology 1993; 4: 292-298.
19. Lange PH, Lightner DJ, Medini E, Reddy PK, Vessella R. The effect of radiation therapy after radical prostatectomy in patients with elevated prostate specific antigen levels. J Urol 1990; 144: 927-932.
20. Schild SE, Grado GL, Buskirk SJ, Robinow JS, Wong WW, Frick LM, Ferrigni RG. The use of radiotherapy for patients with isolated elevation of serum PSA levels following radical prostatectomy. Mayo Clin Proc in press.
21. McCarthy JF, Catalona WJ, Hudson MA. Effect of radiation therapy on detectable serum prostate specific antigen levels following radical prostatectomy: early versus delayed treatment. J Urol 1994; 151: 1575-1578.
22. Partin AW, Pound CP, Clemens JQ, Epstein JI, Walsh PC. Serum PSA after anatomic radical prostatectomy. Urol Clin North Am 1993; 20: 713-725.
23. Freeman JA, Liesovsky G, Cook DW, Petrovitch Z, Chen S, Groshen S, Skinner DG. Radical retropubic prostatectomy and postoperative radiation for pathological stage C prostate cancer 1976-1989: intermediate findings. J Urol 1993; 149: 1029.
24. Dumas F, Eschwege P, Blanchet P, et al. Enhanced detection of hematogenous circulating cells in patients with prostate adenocarcinoma using prostate specific membrane antigen (PSM)- based nested reverse transcriptase polymerase chain reaction. J Urol 155;780, 1996 (abstract).

**This article should be referenced as follows:**

Ziada, A.M. and Crawford, E.D. (2004) Radiation therapy after radical prostatectomy. *TheScientificWorldJOURNAL* **4 (S1)**, 377–381.

**Handling Editor:**

Anthony Atala, Principle Editor for *Urology* — a domain of *TheScientificWorldJOURNAL.*
